# Supplementary material for: Genomic and Temporal Trends in Canine ExPEC Reflect Those of Human ExPEC
Source: Microbiol Spectr. 2022 Jun 8;10(3):e01291-22. doi: 10.1128/spectrum.01291-22 (PMC9241711; doi:10.1128/spectrum.01291-22)
Supplement: Supplemental file 1 — Supplemental material. Download spectrum.01291-22-s0001.pdf, PDF file, 5.6 MB [file spectrum.01291-22-s0001.pdf]

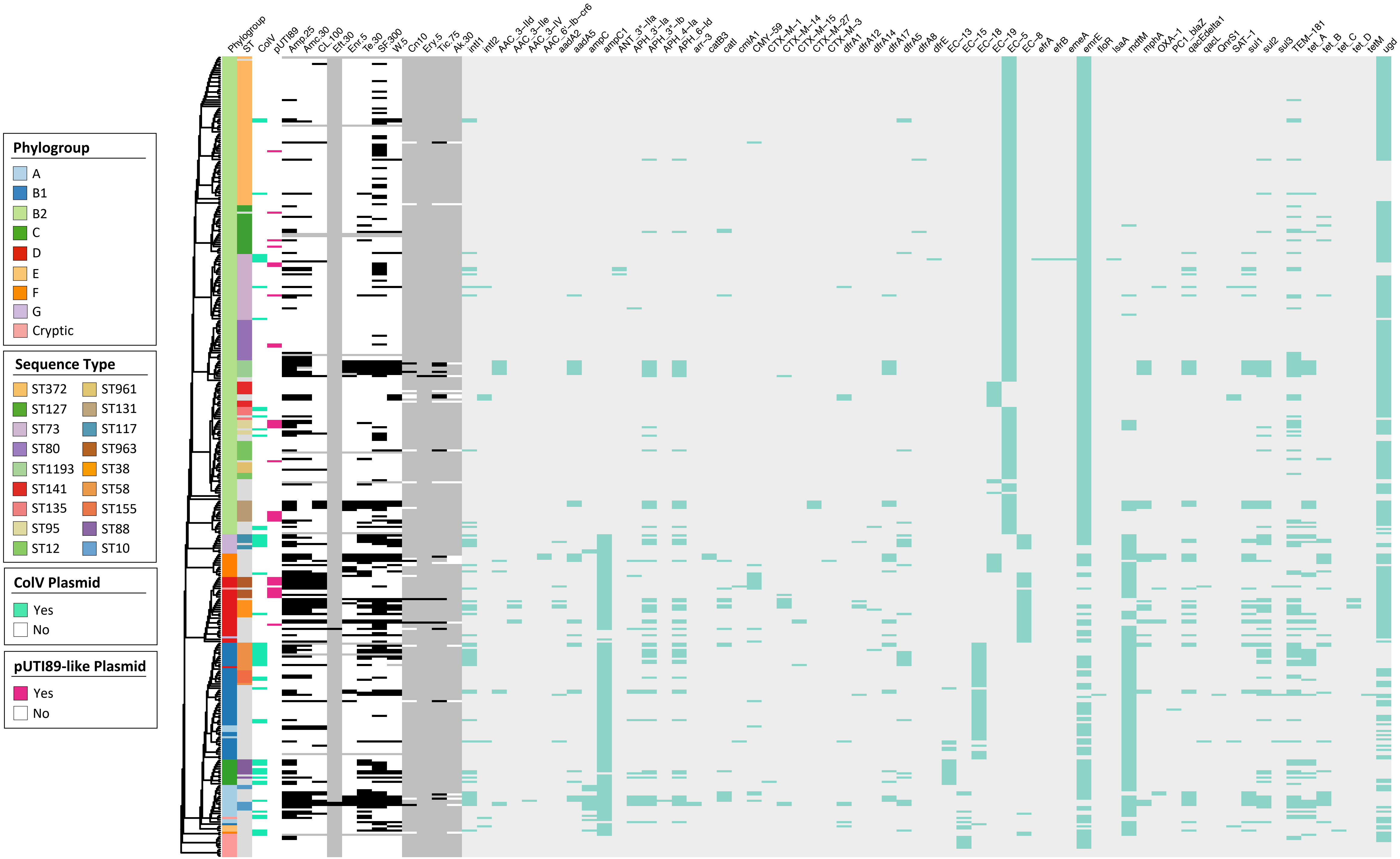
Phylogroup
A
B1
B2
C
D
E
F
G
Cryptic
Sequence Type
ST372
ST961
ST127
ST131
ST73
ST117
ST80
ST963
ST1193
ST38
ST141
ST58
ST135
ST155
ST95
ST88
ST12
ST10
ColV Plasmid
Yes
No
pUTI89-like Plasmid
Yes
No
Phylogroup
ST
ColV
pUTI89
Amp.25
Amc.30
CL.100
Eft.30
Enr.5
Te.30
SF300
W.5
Cn10
Ery.5
Tic.75
Ak.30
intI1
intI2
AAC_3–IId
AAC_3–IIe
AAC_3–IV
AAC_6'–Ib–cr6
aadA2
aadA5
ampC
ampC1
ANT_3''–IIa
APH_3'–Ia
APH_3''–Ib
APH_4–Ia
APH_6–Id
arr–3
catB3
catI
cmlA1
CMY–59
CTX–M–1
CTX–M–14
CTX–M–15
CTX–M–27
CTX–M–3
dfrA1
dfrA12
dfrA14
dfrA17
dfrA5
dfrA8
dfrE
EC–13
EC–15
EC–18
EC–19
EC–5
EC–8
efrA
efrB
emeA
emrE
floR
lsaA
mdtM
mphA
OXA–1
PC1_blaZ
qacEdelta1
qacL
QnrS1
SAT–1
sul1
sul2
sul3
TEM–181
tet_A
tet_B
tet_C
tet_D
tetM
ugd

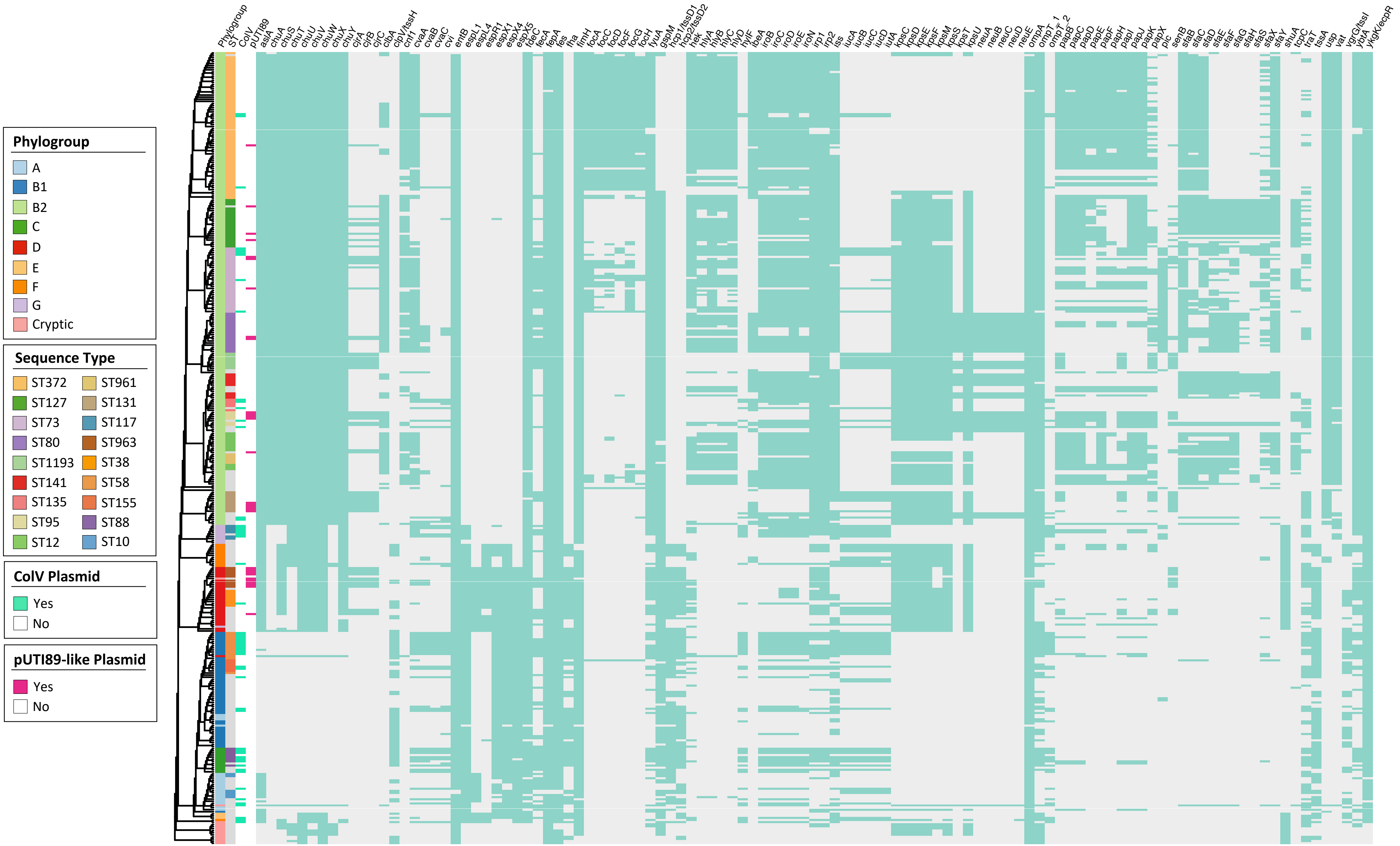

Phylogroup
ST
ColV
pUTI89
aslA
chuA
chuS
chuT
chuU
chuV
chuW
chuX
chuY
cjrA
cjrB
cjrC
clbA
clpV/tssH
cnf1
cvaA
cvaB
cvaC
cvi
entB
espL1
espL4
espR1
espX1
espX4
espX5
fdeC
fecA
fepA
fes
fha
fimH
focA
focC
focD
focF
focG
focH
fyuA
gspM
hcp1/tssD1
hcp2/tssD2
hek
hlyA
hlyB
hlyC
hlyD
hlyF
ibeA
iroB
iroC
iroD
iroE
iroN
irp1
irp2
iss
iucA
iucB
iucC
iucD
iutA
kpsC
kpsD
kpsE
kpsF
kpsM
kpsS
kpsT
kpsU
neuA
neuB
neuC
neuD
neuE
ompA
ompT_1
ompT_2
papB
papC
papD
papE
papF
papH
papI
papJ
papK
papX
pic
senB
sfaB
sfaC
sfaD
sfaE
sfaF
sfaG
sfaH
sfaS
sfaX
sfaY
shuA
tcpC
traT
tssA
usp
vat
vgrG/tssI
ybtA
ykgK/ecpR
Phylogroup
A
B1
B2
C
D
E
F
G
Cryptic
Sequence Type
ST372
ST961
ST127
ST131
ST73
ST117
ST80
ST963
ST1193
ST38
ST141
ST58
ST135
ST155
ST95
ST88
ST12
ST10
ColV Plasmid
Yes
No
pUTI89-like Plasmid
Yes
No

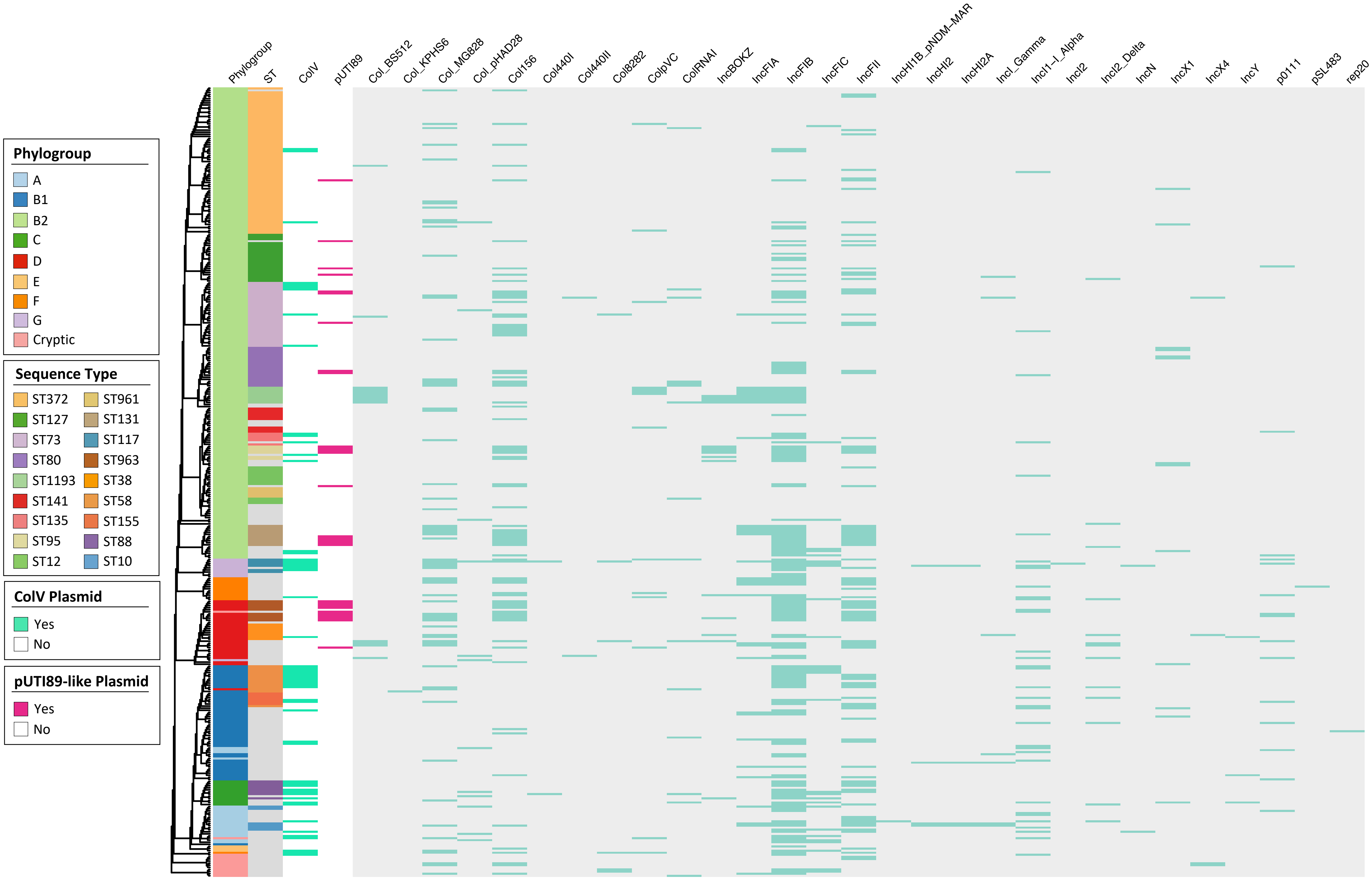

Phylogroup
A
B1
B2
C
D
E
F
G
Cryptic
Sequence Type
ST372
ST127
ST73
ST80
ST1193
ST141
ST135
ST95
ST12
ST961
ST131
ST117
ST963
ST38
ST58
ST155
ST88
ST10
ColV Plasmid
Yes
No
pUTI89-like Plasmid
Yes
No
Phylogroup
ST
ColV
pUTI89
Col_BS512
Col_KPHS6
Col_MG828
Col_pHAD28
Col156
Col440I
Col440II
Col8282
ColpVC
ColRNAI
IncB/O/K/Z
IncFIA
IncFIB
IncFIC
IncFII
IncHI1B_pNDM-MAR
IncHI2
IncHI2A
IncI_Gamma
IncI1-I_Alpha
IncI2
IncI2_Delta
IncN
IncX1
IncX4
IncY
p0111
pSL483
rep20

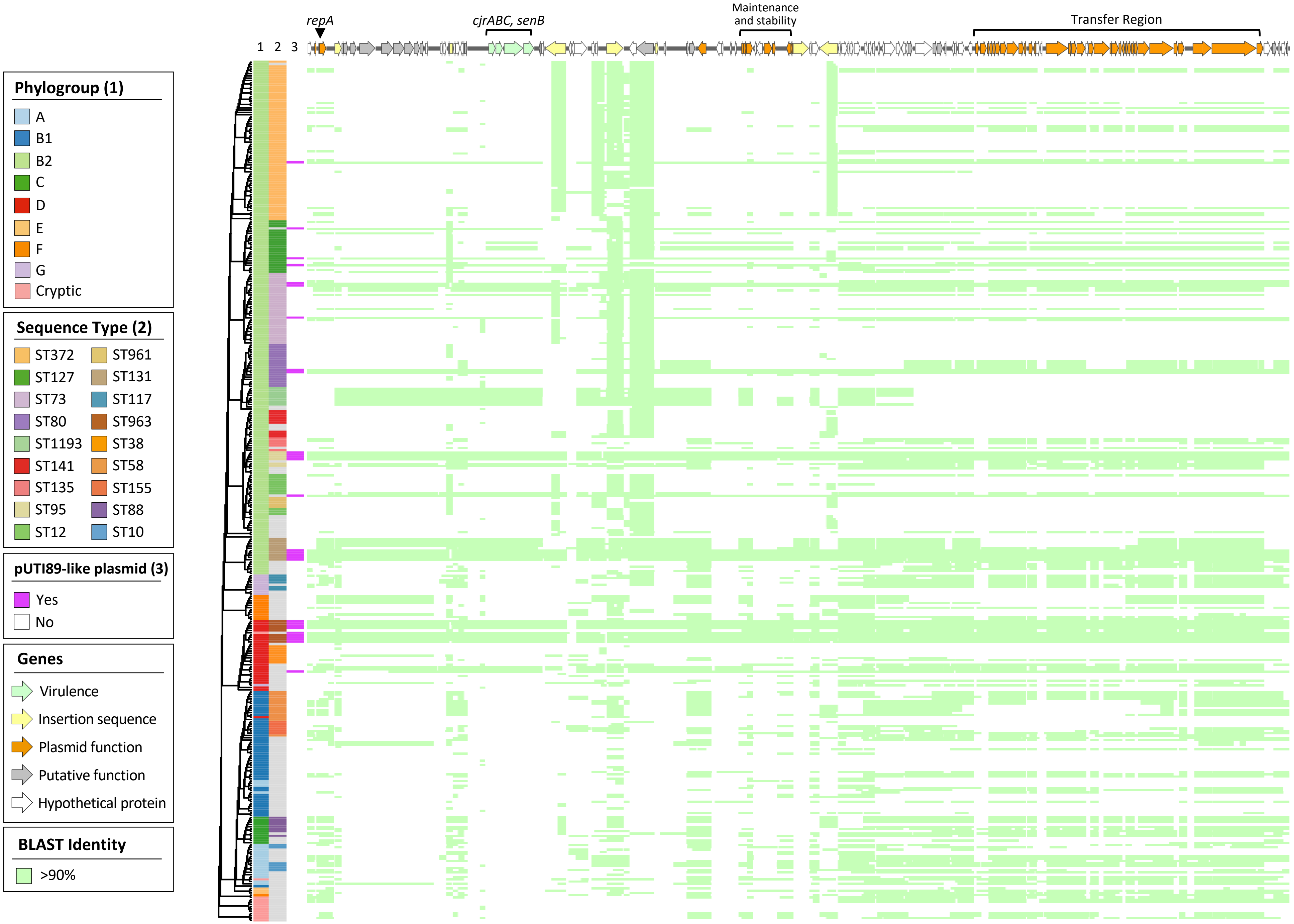
repA
cjrABC, senB
Maintenance
and stability
Transfer Region
1
2
3
Phylogroup (1)
A
B1
B2
C
D
E
F
G
Cryptic
Sequence Type (2)
ST372
ST961
ST127
ST131
ST73
ST117
ST80
ST963
ST1193
ST38
ST141
ST58
ST135
ST155
ST95
ST88
ST12
ST10
pUTI89-like plasmid (3)
Yes
No
Genes
Virulence
Insertion sequence
Plasmid function
Putative function
Hypothetical protein
BLAST Identity
>90%

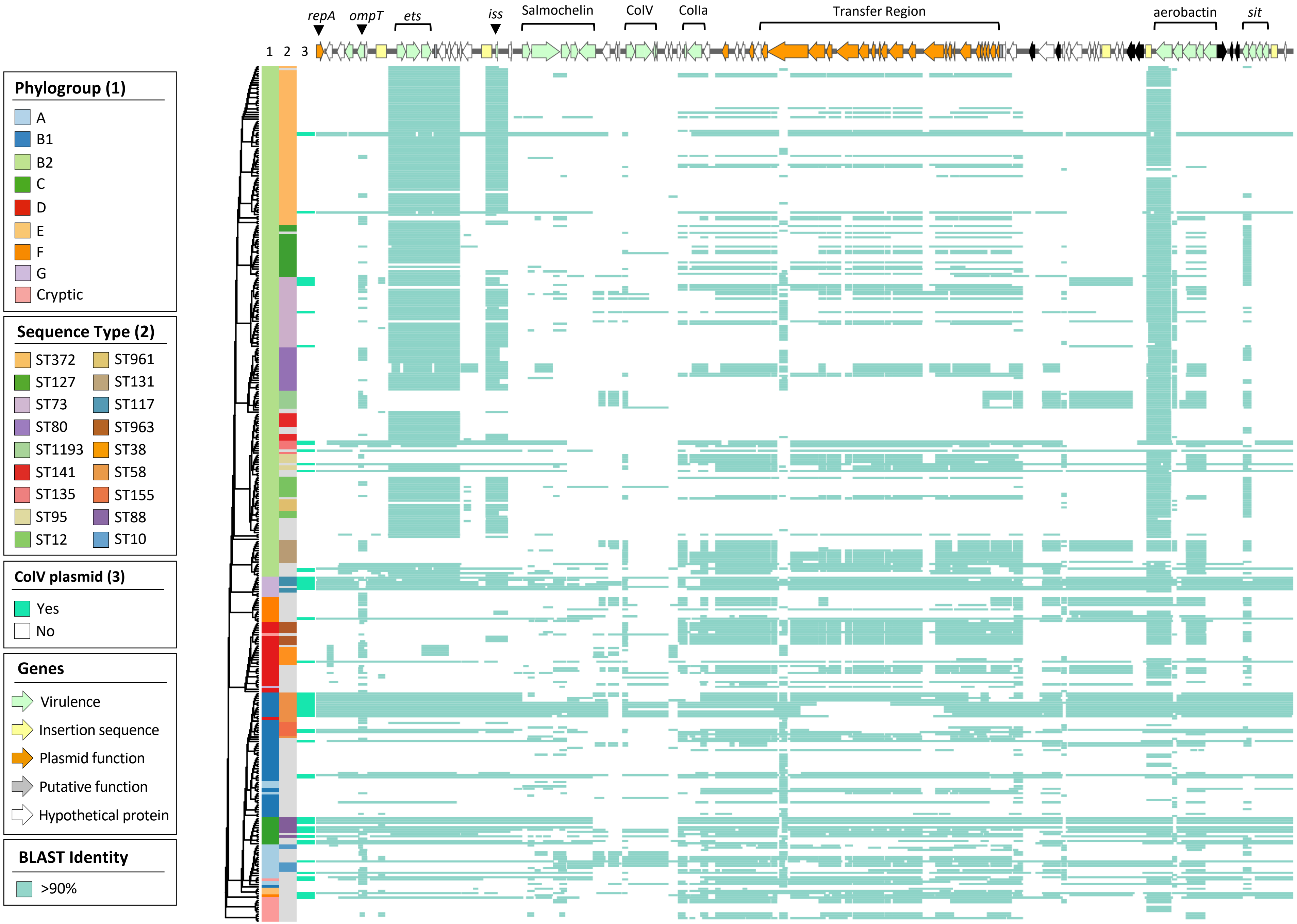
repA
ompT
ets
iss
Salmochelin
ColV
Colla
Transfer Region
aerobactin
sit
1
2
3
Phylogroup (1)
A
B1
B2
C
D
E
F
G
Cryptic
Sequence Type (2)
ST372
ST961
ST127
ST131
ST73
ST117
ST80
ST963
ST1193
ST38
ST141
ST58
ST135
ST155
ST95
ST88
ST12
ST10
ColV plasmid (3)
Yes
No
Genes
Virulence
Insertion sequence
Plasmid function
Putative function
Hypothetical protein
BLAST Identity
>90%

Figure S1. Antimicrobial resistance heatmap. Binary heatmap of antimicrobial resistance phenotype (black/white) and resistance gene presence/absence (green/grey) aligned to core gene phylogeny with metadata.

Figure S2. Virulence-associated gene heatmap. Binary heatmap of virulence-associated gene presence/absence (green/grey) aligned to core gene phylogeny with metadata.

Figure S3. Plasmid-associated gene heatmap. Binary heatmap of plasmid-associated gene presence/absence (green/grey) aligned to core gene phyloge`ny with metadata.

Figure S4. Sequence mapping to pUTI89. Heatmap of nucleotide identity across 100bp segments of the pUTI89 backbone. Metadata for phylogroup and ST are shown. Pink metadata bars show isolates matching at least 90% of the backbone and/or carrying F29:A-:B10 RST.

Figure S5. Sequence mapping to pCERC4. Heatmap of nucleotide identity across 100bp segments of the pCERC4 backbone. Metadata for phylogroup and ST are shown. Green bars show isolates determined to carry a ColV plasmid based on the Liu criteria (see Methods).
